# Pediatric Microbiota: Evidence and Future Perspectives—A Conference Report

**DOI:** 10.3390/microorganisms14102130

**Published:** 2026-09-22

**Authors:** Sandra Ramos, Juan Carlos Ayala-García, Ana Isabel Burguete-García, José Francisco Cadena-León, Amapola De Sales-Millán, Jaime García-Mena, Leopoldo Santos-Argumedo, Miguel A. Vázquez-Moreno, José Antonio Velázquez-Aragón

**Affiliations:** 1 Experimental Oncology Laboratory, National Pediatric Institute, Mexico City 04530, Mexico; sramosa@pediatria.gob.mx; 2 National Institute of Public Health, Center for Infectious Disease Research, Cuernavaca 62100, Mexico; carlos.ayala@insp.edu.mx (J.C.A.-G.); aburguete@insp.mx (A.I.B.-G.); 3 Gastroenterology and Nutrition Service, National Pediatric Institute, Mexico City 04530, Mexico; pcaden60@hotmail.com; 4 Division of Biological and Health Sciences, Autonomous Metropolitan University-Lerma, Lerma 52006, Mexico; 2212803115@correo.ler.uam.mx; 5 Centro de Investigación y Estudios Avanzados (CINVESTAV), Department of Genetics and Molecular Biology, Av. Instituto Politécnico Nacional 2508, Mexico City 07360, Mexico; jgmena@cinvestav.mx; 6 Centro de Investigación y Estudios Avanzados (CINVESTAV), Department of Molecular Biomedicine and Center for Research on Aging, Calz. de los Tenorios 235, Granjas Coapa, Tlalpan, Mexico City 14330, Mexico; lesantos@cinvestav.mx; 7 Unidad de Investigación Médica en Bioquímica, Centro Médico Nacional Siglo XXI, Instituto Mexicano del Seguro Social, Mexico City 06720, Mexico

**Keywords:** pediatric microbiome, gut microbiota, microbiota–gut–brain axis, breastfeeding, neonatal immunity, short-chain fatty acids, metabolic health, autism spectrum disorder, sleep

## Abstract

During early life, the human microbiota contributes to the development of immune, metabolic, and neurological functions. Antibiotic exposure, inflammation, and dietary changes can alter microbial community structure and function, with potential implications for pediatric health. In April 2026, the symposium “Pediatric Microbiota: Evidence and Future” was held in Mexico City, bringing together experts to discuss current advances in pediatric microbiome research. This conference report summarizes the major topics and perspectives presented during the symposium. Several presentations examined how microorganisms and microbial metabolites are transferred from mother to infant. The speakers also discussed the contribution of colostrum immunoglobulins, particularly IgA, to early microbial colonization. Sessions reviewed evidence supporting probiotic use in pediatric gastrointestinal disorders and explored the microbiota–gut–brain axis in autism spectrum disorder and sleep regulation. In addition, speakers discussed the interactions among diet, starch digestion, microbial fermentation, and metabolic health, and their associations with obesity, insulin resistance, and cardiometabolic risk. Important knowledge gaps were identified in pediatric oncology, where evidence remains limited regarding the microbiome’s influence on cancer susceptibility, immune responses, treatment outcomes, and therapy-related toxicities. Participants agreed that the field would benefit from longitudinal studies in well-characterized pediatric cohorts, particularly in Latin America, as well as from studies examining biological mechanisms through multi-omics approaches. This research could eventually inform precision nutrition and microbiome-based interventions, but the evidence discussed at the symposium was not considered sufficient to support their routine clinical use.

## 1. Introduction

The gut microbiome is a complex microbial ecosystem that interacts with host physiology throughout life. These interactions are relevant to both normal development and disease. Childhood is a particularly important period because microbial communities develop while the immune, metabolic, gastrointestinal, and nervous systems are still maturing. Changes during this period may affect both childhood health and later disease risk [1,2,3,4].

The pediatric microbiome is shaped by a wide range of maternal, perinatal, dietary, environmental, and host-related factors, and its composition and function continue to change throughout childhood [2,3,5]. Despite the growing number of studies, many aspects of microbiome development during childhood remain poorly characterized. Longitudinal studies are needed to determine how pediatric microbial communities change over time and how these changes relate to clinical outcomes [1,2,3,4,6].

Microbiome alterations have also been studied in chronic inflammatory diseases and cancer. In these settings, researchers are examining possible associations with disease development, progression, and treatment response.

In this context, the symposium Pediatric Microbiota: Evidence and Future, held in Mexico City in April 2026, brought together experts from different disciplines to discuss current evidence and emerging perspectives in pediatric microbiome research. The presentations addressed complementary aspects of the field, ranging from early-life and clinical applications to diet–microbiome interactions, metabolic health, neurodevelopmental disorders, and sleep. Across the presentations, speakers noted growing interest in microbiome-based interventions but also emphasized that pediatric evidence remains insufficient for many clinical applications [7,8,9,10,11].

## 2. Preparation of the Conference Report

The symposium Pediatric Microbiota: Evidence and Future took place on 14 and 15 April 2026, at the Instituto Nacional de Pediatría (INP) in Mexico City, Mexico (Figure S1). The event was conducted in a hybrid format and brought together approximately 200 participants, with half attending in person and the remainder joining remotely.

Attendees represented several clinical and research fields, including pediatric gastroenterology, immunology, nutrition, molecular biology, neurodevelopment, and microbiome research. It was organized by Sandra Ramos and José Velázquez-Aragón from the Experimental Oncology Laboratory at the National Institute of Pediatrics (INP), with the participation of invited speakers and collaborators from multiple academic, clinical, and research institutions. The audience included pediatric healthcare professionals, researchers, graduate students, and trainees with an interest in pediatric microbiome research and its clinical and translational applications.

The program included seven invited lectures organized into three thematic sessions. Speakers were invited on the basis of their expertise in the principal topics covered by the symposium: early-life microbiome development, clinical applications, diet and metabolic health, neurodevelopment, and the microbiota–gut–brain axis.

The presentations included a combination of published evidence, expert interpretation, and, in some cases, findings derived from the speakers’ own research programs. Importantly, the scientific program was not designed exclusively for the presentation of unpublished original research. Additional speakers and presentations were included; however, this conference report is limited to contributions from speakers who agreed to participate in the preparation of the manuscript.

After the symposium, each participating speaker provided a written summary of his or her presentation for inclusion in this report. Each section reflects the content of the corresponding speaker’s summary, and the references cited within each section generally correspond to the scientific literature selected by the speaker to support or discuss the material presented. Additional references were incorporated during manuscript revision where necessary to provide relevant scientific context or address specific points raised during peer review; these additions did not alter the scientific content of the presentations.

During manuscript preparation, the submitted summaries were edited for clarity, consistency, and integration into the overall narrative of the conference report, while preserving the original scientific content of the presentations. Each corresponding section was subsequently circulated to the respective speaker for review and approval. Selected key references discussed during the symposium, together with their study design, methodology, main findings, and limitations, are summarized in Supplementary Table S1.

This conference report records the topics presented at the symposium and summarizes the speakers’ main points. It is not a systematic review, consensus statement, or clinical guideline. Its scope is limited to the participating speakers’ contributions and to the literature used to support their presentations. The strength and type of evidence also differed among the topics discussed.

## 3. Conference Sections

The symposium was organized into three thematic sessions. Each presentation addressed a specific aspect of pediatric microbiome research, allowing the discussion to progress from early-life microbial establishment and clinical applications to metabolic and neurodevelopmental implications. Table 1 provides an overview of the scientific program represented in this Conference Report.

### 3.1. Microbiome During Lactation, Clinical Pediatrics and Immune Regulation

#### 3.1.1. Probiotics in Pediatrics: From Evidence to Clinical Practice

The presentation reviewed microbiome-directed interventions used in pediatric practice, with particular attention to probiotics. Among these interventions, probiotics represent one of the most extensive approaches, particularly for gastrointestinal disorders associated with altered intestinal microbial composition and function [12]. Current evidence indicates that probiotics are live microorganisms that, when administered in adequate amounts, confer a health benefit; however, their clinical effects depend on the specific strain, dose, and disease being treated [13,14].

A central concept of the discussion was the role of the intestinal microbiota in maintaining gastrointestinal and systemic homeostasis through its contributions to nutrient metabolism, intestinal barrier integrity, immune regulation, and host metabolism. Alterations in microbial composition and function have been associated with gastrointestinal, allergic, metabolic, autoimmune, and inflammatory diseases. The first 1000 days of life were highlighted as a critical developmental period during which factors such as delivery mode, breastfeeding, antibiotic exposure, and maternal health shape microbiome development and may influence long-term pediatric health.

Diet was identified as an important determinant of probiotic efficacy. Fiber-rich diets, including resistant starches and fermentable carbohydrates, enhance microbial recovery by promoting bacterial growth, improving intestinal transit, increasing the production of short-chain fatty acids (SCFAs), and creating a metabolic environment favorable for beneficial microorganisms [12].

The speaker described several mechanisms of probiotic activity, including pathogen exclusion, production of antimicrobial compounds and SCFAs, modulation of immune responses, and preservation of epithelial barrier function. These mechanisms indicate that the effects of probiotics depend on their interactions with resident microorganisms and host tissues; probiotics should not be viewed simply as replacements for absent bacteria [15].

From a clinical perspective, the updated position paper of the European Society for Paediatric Gastroenterology, Hepatology and Nutrition (ESPGHAN) was discussed as a framework for evaluating probiotic use in selected pediatric gastrointestinal conditions. Importantly, the ESPGHAN Working Group did not issue a recommendation for probiotics as a general category; recommendations were formulated only for specific strains or combinations when sufficient evidence from randomized controlled trials was available [14]. The recommendations therefore depend on the strain, indication, dose, population, and clinical context, and should not be extrapolated to other probiotic preparations (Table 2).

The evidence reviewed supports the use of particular probiotic strains in a limited number of pediatric gastrointestinal conditions. However, their clinical application should remain indication and strain-specific, and further well-designed clinical trials are needed to clarify efficacy, safety, treatment response, and the populations most likely to benefit.

The next presentation shifted the focus from probiotic interventions to maternal immune factors involved in the establishment of the neonatal microbiota.

#### 3.1.2. The Specialized Role of Maternal IgA Subclasses in the Configuration of the Microbiota and Neonatal Immunity

The presentation examined how the maternal IgA1 and IgA2 subclasses contribute to neonatal mucosal immunity and interact with the developing gut microbiota. Birth represents a critical developmental transition during which newborns are abruptly exposed to a diverse microbial environment, making the early postnatal period particularly important for the development of host–microbe interactions and protection against infection. During the first days after delivery, colostrum provides a rich source of maternal immune factors, including cytokines, growth factors, and immunoglobulins, among which secretory IgA (sIgA) plays a central role in mucosal protection and microbial regulation.

A key aspect of the discussion was the functional diversity of the two human IgA subclasses, IgA1 and IgA2, which differ in their structure, tissue distribution, and antigen recognition profiles. The immunological history of the mother during pregnancy may influence the composition of colostrum [16]. In particular, infectious episodes at different mucosal sites may be associated with distinct IgA1 and IgA2 responses, while the chemical composition of microbial antigens may further influence the subclass-specific antibody repertoire present in colostrum [17].

Evidence derived from studies of *Helicobacter pylori* supports a potential protective role of maternal IgA during lactation. Colostrum IgA1 antibodies can recognize *H. pylori* antigens and interfere with bacterial virulence mechanisms, suggesting a potential role for maternal antibodies in limiting early-life microbial colonization and infection [18].

Beyond pathogen protection, maternal immunoglobulins may also contribute to the selective shaping of the neonatal gut microbiota. Maternal antibodies in colostrum display differential binding patterns toward bacterial communities, with IgA2 and IgM preferentially binding Proteobacteria, whereas IgG is more frequently associated with Firmicutes [19]. Furthermore, IgA2-coated bacteria in neonatal feces have been associated with specific microbial communities and functional pathways related to carbohydrate utilization, epithelial colonization, and folate biosynthesis [20]. These observations indicate that maternal antibodies bind selectively to components of the neonatal microbiota. The developmental consequences of this selective binding remain to be established.

The transfer of maternal immunoglobulins provides passive mucosal protection and may also affect which microorganisms colonize the infant intestine during early life [19,20]. These findings highlight colostrum not only as an essential source of nutrition and passive immunity but also as an important biological mediator of neonatal immune maturation and microbiome development.

These observations underscore the role of maternal immunoglobulins in neonatal immune development and microbial regulation. The following presentation addressed the transfer of maternal microorganisms and milk-associated components to the infant.

#### 3.1.3. Vertical Transmission of Microbiota and Metabolites During Lactation

Dr. Jaime García-Mena presented an overview of current evidence on maternal transmission of the microbiota from birth through lactation, integrating insights from his research experience. The speaker proposed a framework in which human physiology reflects interactions among the nuclear genome, the mitochondrial genome, and the microbiome. Unlike the two genomes, the microbiome is acquired through several ecological routes and changes substantially during early life. Within this framework, maternal microorganisms provide some of the first microbial populations and functions encountered by the infant.

Particular attention was given to the diversity of maternal microbial sources involved in early-life colonization. Through close contact with the newborn, mothers may transmit microorganisms from multiple anatomical sites, including the vagina, gastrointestinal tract, skin, and oral cavity. Human milk represents an additional and biologically important route of microbial exposure, as breastfed infants are repeatedly exposed to milk-associated microorganisms during lactation. Some of these early colonizers may establish persistent associations within the developing infant gut ecosystem, contributing to microbiome maturation during early life.

The speaker also reviewed evidence for the proposed entero-mammary pathway, through which selected maternal gut microorganisms may reach the mammary gland and subsequently be transferred through breast milk. This mechanism may facilitate the transmission of microorganisms adapted to the maternal environment and contribute to the establishment of the neonatal gut microbiota. In addition to microorganisms, human milk contains a diverse array of bioactive metabolites and other compounds that can support microbial growth, intestinal maturation, and immune development in the newborn.

Available evidence suggests that maternal–infant microbial transmission is a complex dynamic process involving multiple microbial sources and biological pathways. Understanding how maternal microorganisms and milk-derived metabolites interact with the developing infant microbiome may yield important insights into early-life microbial colonization, immune maturation, and long-term health. Studies that follow mother–infant pairs over time and analyze maternal microbiota, breast milk, and infant samples together could help determine how maternal microorganisms are transferred and whether they persist in the infant gut.

Dietary patterns, lifestyle, gastrointestinal symptoms, obesity, and gut microbial alterations frequently overlap in pediatric populations. These relationships are likely bidirectional and influenced by multiple host and environmental determinants, making it difficult to attribute metabolic or gastrointestinal outcomes to microbiome alterations alone. Consistent with this perspective, the recent literature has emphasized the need to consider diet, lifestyle, adiposity, gastrointestinal function, and microbial ecology within an integrated framework rather than as independent determinants [21].

### 3.2. Diet–Microbiome Interactions and Metabolic Health

The second session shifted the focus toward the interactions between the gut microbiota, dietary factors, and metabolic health during childhood. The presentations approached these relationships from complementary perspectives, ranging from the broader role of microbiota in metabolic health to the specific contribution of starch digestion and microbial fermentation.

#### 3.2.1. Microbiota: The Invisible Link Between Diet and Metabolic Diseases

Dr. Ana Isabel Burguete-García presented an overview of the central role of the gut microbiota in human development and metabolic health. The presentation described the gut microbiota as an ecosystem that interacts with the host through neural, endocrine, immune, and metabolic pathways. These interactions begin early in life and remain shaped by environmental exposures, particularly dietary patterns and pharmaceutical interventions [22,23,24].

The speaker discussed how differences in microbial composition and metabolic activity may relate to host health [24,25]. The speaker noted that diet, medication use, age, and host characteristics can influence both microbial composition and function, which may partly explain the variation observed across studies. Some metabolic disorders have been associated with a lower abundance of SCFA-producing bacteria, particularly butyrate producers, as well as with impaired intestinal barrier and immune functions [24,26]. These alterations may contribute to metabolic endotoxemia and chronic low-grade inflammation, which are recognized mechanisms associated with obesity, metabolic syndrome, and other metabolic disorders [22,27,28].

Diet exerts a profound influence on microbial composition and function. Dietary patterns rich in fruits, vegetables, legumes, healthy fats, and fiber promote microbial diversity and support beneficial microbial communities, including microorganisms associated with SCFAs production [23,24]. In contrast, diets characterized by high consumption of ultra-processed foods, saturated fats, and simple sugars are associated with microbial imbalance and inflammation [23,24,27,29]. Studies in pediatric populations have further highlighted associations between dietary-dependent alterations in the gut microbiota and childhood obesity, supporting the relevance of diet–microbiota interactions during early development [29].

The Mediterranean diet represents an example of a dietary pattern capable of promoting the abundance of beneficial genera such as *Lactobacillus*, *Akkermansia*, and *Bifidobacterium*, while potentially reducing oxidative stress and inflammatory processes [23,24]. Dietary fiber and prebiotic substrates are of particular interest because they can be fermented by intestinal microorganisms to generate SCFAs, including butyrate, with important effects on epithelial barrier integrity and immune regulation [22,24].

These findings underscored the importance of promoting healthy dietary habits from childhood, including adequate fiber intake, consumption of prebiotic-containing foods, and regular physical activity [23,24,29]. Dietary interventions may alter the gut microbiota, but their effectiveness in preventing or treating pediatric metabolic disease requires further clinical evaluation. In this context, emerging evidence suggests that interactions between gut microbiota composition, adiposity, and inflammatory pathways may contribute to metabolic risk, including through associations between intestinal microbiota and waist circumference [28]. Nevertheless, further research is needed to better understand the complex interactions among diet, microbial ecology, metabolic function, and host health across the lifespan [22,24,28].

The next presentation focused specifically on starch digestion, microbial fermentation, and metabolic risk in Mexican children.

#### 3.2.2. Starch Digestion and Gut Microbiota in Mexican Children: Metabolic Implications for the Risk of Insulin Resistance

The presentation examined the relationships among starch digestion, gut microbiota composition, and cardiometabolic risk in Mexican children. This topic is clinically relevant in Mexico because childhood obesity and insulin resistance are common and may be influenced by dietary, genetic, and microbial factors.

Genetic variation in salivary and pancreatic amylases represents an important factor influencing starch digestion efficiency. Copy number variation in the *AMY1* gene family and the pancreatic amylase genes *AMY2A* and *AMY2B* have been associated with interindividual differences in starch digestion efficiency. Particular attention was given to findings from a previous study in Mexican children that evaluated copy number variation in *AMY1A* and *AMY2A* and its relationship with metabolic traits [30].

Specific intestinal microorganisms can ferment resistant starch and produce SCFAs, including acetate, propionate, and butyrate [31,32]. These microbial metabolites contribute to intestinal barrier integrity, glucose metabolism, energy homeostasis, and the modulation of inflammatory pathways [33]. Consequently, differences in starch digestion and microbial fermentation may influence metabolic phenotypes and potentially contribute to susceptibility to obesity and insulin resistance during childhood.

The relationship between host genetics, dietary starch intake, and the gut microbiota is highly complex and remains incompletely understood. Longitudinal and functional studies integrating host genetic variation, dietary exposures, microbial composition, and metabolic activity are needed to better understand these interactions. Clarifying these interactions could help determine whether dietary recommendations based on starch digestion or microbial fermentation are feasible and clinically useful in children.

Collectively, these observations illustrate the complexity of interactions among host characteristics, dietary substrates, and microbial function. The symposium then moved from metabolic health to another area in which these interactions have received increasing attention: neurodevelopmental health and the microbiota–gut–brain axis.

### 3.3. The Microbiota–Gut–Brain Axis in Pediatric Health: Neurodevelopmental Disorders, Behavior, and Sleep

The third session addressed the relationship between the gut microbiota and neurological and behavioral health. The presentations focused on two related areas, Autism Spectrum Disorder and sleep, extending the discussion toward neurodevelopmental and behavioral aspects of pediatric health.

#### 3.3.1. The Microbiome–Gut–Mucosal–Immune System–Brain Axis and Autism Spectrum Disorder

The gut microbiome participates in bidirectional signaling among the intestine, mucosal immune system, and central nervous system. Gut microbiome development coincides with major stages of immune and neurological maturation during early life. Whether changes in the microbiome directly affect these developmental processes remains under investigation. In children with ASD, gastrointestinal symptoms and alterations in the gut microbiome have been consistently reported [1,4].

Particular attention was given to the microbiome–gut–mucosal–immune system–brain axis as a framework for understanding the complex interactions between intestinal microorganisms and neurodevelopment. This model emphasizes that microbial alterations may influence host physiology through interconnected mechanisms involving intestinal barrier function, immune signaling, microbial metabolites, and neuroactive compounds. Through these pathways, the gut microbiome may participate in bidirectional communication with the central nervous system and potentially influence behavioral and neurological processes associated with ASD [4].

Available evidence suggests differences in gut microbial composition and function among children with ASD, while emphasizing that findings have varied across studies and populations [34,35]. These differences may be influenced by age, sex, diet, symptom severity, and other environmental and host-related factors. Research from Dr. Amapola De Sales-Millán and colleagues has contributed to the characterization of gut microbiome composition and functional metabolic profiles in children with ASD, including potential sex-related differences and alterations in microbial metabolic pathways [34]. These findings support the concept that ASD-associated microbiome alterations may involve not only changes in microbial taxonomy but also modifications in microbial ecosystem function and host–microbe metabolic interactions.

The speaker also discussed the clinical relevance of gastrointestinal symptoms in children with ASD, including constipation, diarrhea, abdominal pain, and altered bowel habits [36]. Although the directionality and causality of the relationships among gastrointestinal dysfunction, microbiome alterations, and ASD manifestations remain uncertain, accumulating evidence supports the importance of considering gastrointestinal health as part of comprehensive pediatric care for children with ASD [1,36].

The presentation reviewed probiotics, prebiotics, and synbiotics as possible approaches to modifying the gut microbiota in children with ASD. Clinical studies and meta-analyses have reported promising but heterogeneous findings, highlighting the importance of identifying specific microbial targets, defining appropriate intervention strategies, and considering individual characteristics that may influence treatment response [37]. Recent studies evaluating precision microbial interventions further suggest that microbiome modulation may improve selected clinical outcomes, although robust evidence regarding effects on core ASD symptoms remains limited [7].

The evidence presented indicates that studies of ASD should assess microbial functions and host responses in addition to reporting differences in individual bacterial taxa. Future research should integrate longitudinal microbiome profiling with clinical, nutritional, metabolic, and immunological data to clarify the mechanisms linking intestinal microbial alterations with ASD phenotypes. These studies should first determine which microbiome findings are reproducible and whether any of the observed associations are causal. Only then could they provide a basis for individualized interventions.

The observations highlight the complexity of interactions among microbial ecology, gastrointestinal function, immunity, and neurodevelopment. The following presentation extended the discussion of the microbiota–gut–brain axis to another aspect of pediatric health, focusing on the relationship between the gut microbiota and sleep.

#### 3.3.2. Microbiota and Sleep Disorders in Pediatric Populations

The presentation reviewed current evidence on the relationship between the gut microbiota and sleep in children. Sleep supports several developmental processes, including synaptic remodeling, myelination, and hormonal regulation. Throughout childhood and adolescence, sleep architecture undergoes dynamic changes regulated by interacting homeostatic and circadian mechanisms [38].

The bidirectional relationship between sleep and gut microbiota has emerged as an important area of investigation. Proposed mechanisms linking the microbiota and sleep include neural, endocrine, immune, and metabolic signaling. However, the contribution of microbiota to sleep regulation in children has not yet been clearly established [39]. Several microbial taxa have been implicated in the production or modulation of neuroactive compounds potentially involved in sleep regulation, including metabolites associated with GABAergic and serotonergic signaling, as well as tryptophan metabolism and melatonin-related pathways [40].

Altered gut microbial profiles have been reported in association with insomnia, fragmented sleep, and obstructive sleep apnea, although these observations do not establish causality. Modern lifestyle factors, such as irregular sleep schedules, excessive screen exposure, late-night eating, and early antibiotic use, may influence both sleep patterns and microbial composition. These observations further support the bidirectional nature of the microbiota–sleep relationship [41].

The presentation concluded that studies of pediatric sleep should account for diet, physical activity, medication use, and other lifestyle factors that can also influence the gut microbiota. A better understanding of the mechanisms linking microbial ecology with sleep regulation may contribute to the development of integrative strategies combining healthy sleep practices, balanced nutrition, physical activity, and lifestyle factors that may influence gut microbial composition and function. Further longitudinal and mechanistic studies are needed to clarify the directionality of these associations and determine whether microbiome-targeted interventions can contribute to improved sleep and broader health outcomes in pediatric populations.

Taken together, these findings illustrate the interconnected nature of microbial, host, environmental, and clinical factors in pediatric health. Across the symposium, complementary aspects of microbiome biology were examined, ranging from early-life microbial establishment and clinical applications to metabolic and neurodevelopmental outcomes (Figure 1).

## 4. Concluding Remarks

The symposium reviewed evidence linking the pediatric gut microbiome with immune maturation, metabolism, and neurodevelopment, particularly during early life. The presentations emphasized that the gut microbiome functions as a dynamic ecosystem whose composition and activity are influenced by maternal factors, nutrition, environmental exposures, and host physiology throughout childhood.

Several speakers emphasized the need to determine how microbial communities develop, respond to disruption, and recover during infancy and childhood. Associations with gastrointestinal, metabolic, immune, and neurodevelopmental outcomes have been reported, but causal relationships remain uncertain. The presentations emphasized the importance of moving beyond descriptive studies toward functional and mechanistic approaches integrating microbial ecology with host biology.

Despite significant progress, important knowledge gaps remain, particularly in pediatric populations where longitudinal studies are still limited and evidence from historically underrepresented regions of the world, such as Latin America, remains scarce. Pediatric oncology was identified as an area requiring additional microbiome research, particularly in relation to treatment response, toxicity, immune regulation, and survivorship. Future symposia should further broaden this perspective by addressing the role of the gut microbiota in pediatric diseases, particularly autoimmune and immune-mediated disorders and cancer, while expanding the discussion of its contribution to metabolic health and related disorders.

The main research priorities identified during the symposium were longitudinal follow-up, mechanistic studies, standardized methods, and collaboration among clinical and laboratory research groups. Translation into preventive or personalized interventions will require prospective clinical validation (Figure 2).

## 5. Limitations

This conference report has several limitations. It provides a narrative account of the symposium based primarily on written summaries submitted by the participating speakers. Consequently, the report does not constitute a systematic review, consensus statement, or clinical guideline. Its scope is restricted to the presentations included in the manuscript and therefore does not encompass the full range of topics within pediatric microbiome research.

The studies discussed by the speakers differed considerably in design, methodology, study population, and quality of evidence. This heterogeneity makes direct comparisons across topics difficult and limits the ability to draw firm conclusions regarding causality or clinical applicability. Therefore, the perspectives presented in this report should be interpreted within the context of the symposium and the evidence reviewed by the respective speakers.

## 6. Recommendations

Based on the discussions presented during the symposium, the following priorities are proposed to advance pediatric microbiome research:

- Establish longitudinal pediatric cohorts spanning pregnancy through childhood to characterize microbiome development and its impact on immune, metabolic, neurodevelopmental, and long-term health outcomes.
- Promote mechanistic and multi-omics studies integrating microbiome composition and function with host genetics, immunity, metabolism, and clinical phenotypes to identify potential causal mechanisms and biomarkers. Such approaches should incorporate longitudinal sampling, standardized methodologies, and appropriate validation, given the substantial intra-individual and inter-individual variability observed in microbiome and multi-omics studies, which may limit reproducibility and the ability to draw firm conclusions [42].
- Include pediatric populations that have been underrepresented in microbiome studies, particularly in Latin America. Multi-omics programs should also support local research capacity, shared infrastructure, and access to analytical expertise. Although multi-omics technologies remain costly and require specialized technical and computational resources, they are essential for gaining a deeper understanding of the complex interactions among the microbiome, host biology, environmental exposures, and pediatric health. Because these analyses are expensive, studies should be designed to produce findings that can eventually be evaluated with simpler and more accessible methods. Multi-omics findings should be validated to determine whether they can identify reproducible mechanisms or biomarkers that are measurable with affordable clinical assays. To make this possible, studies should include representative populations, promote equitable access to data and research opportunities, validate findings across diverse clinical settings, and consider affordability from the early stages of development. Equity should therefore be considered during study design, validation, and implementation so that these technologies do not widen existing disparities in pediatric research and care.
- Prioritize research in historically underrepresented populations, particularly among pediatric cohorts in Latin America, to build population-specific evidence and strengthen the global generalizability of microbiome findings.
- Investigate the maternal–infant microbiome continuum, including the contribution of maternal microbiota, colostrum, breast milk, and early nutrition to microbial colonization and immune development.
- Evaluate microbiome-based interventions, including probiotics, prebiotics, synbiotics, dietary interventions, and precision nutrition, through rigorous, strain-specific clinical trials.
- Expand microbiome research into emerging pediatric fields, particularly pediatric oncology, to clarify how the microbiome shapes cancer biology, modulates treatment response, and contributes to therapy-associated toxicity, and survivorship outcomes.

These priorities emphasize standardized methods, collaboration across disciplines, and the inclusion of diverse pediatric populations. Progress in these areas will be necessary before microbiome findings can be applied consistently in clinical practice.

## Figures and Tables

**Figure 1:**
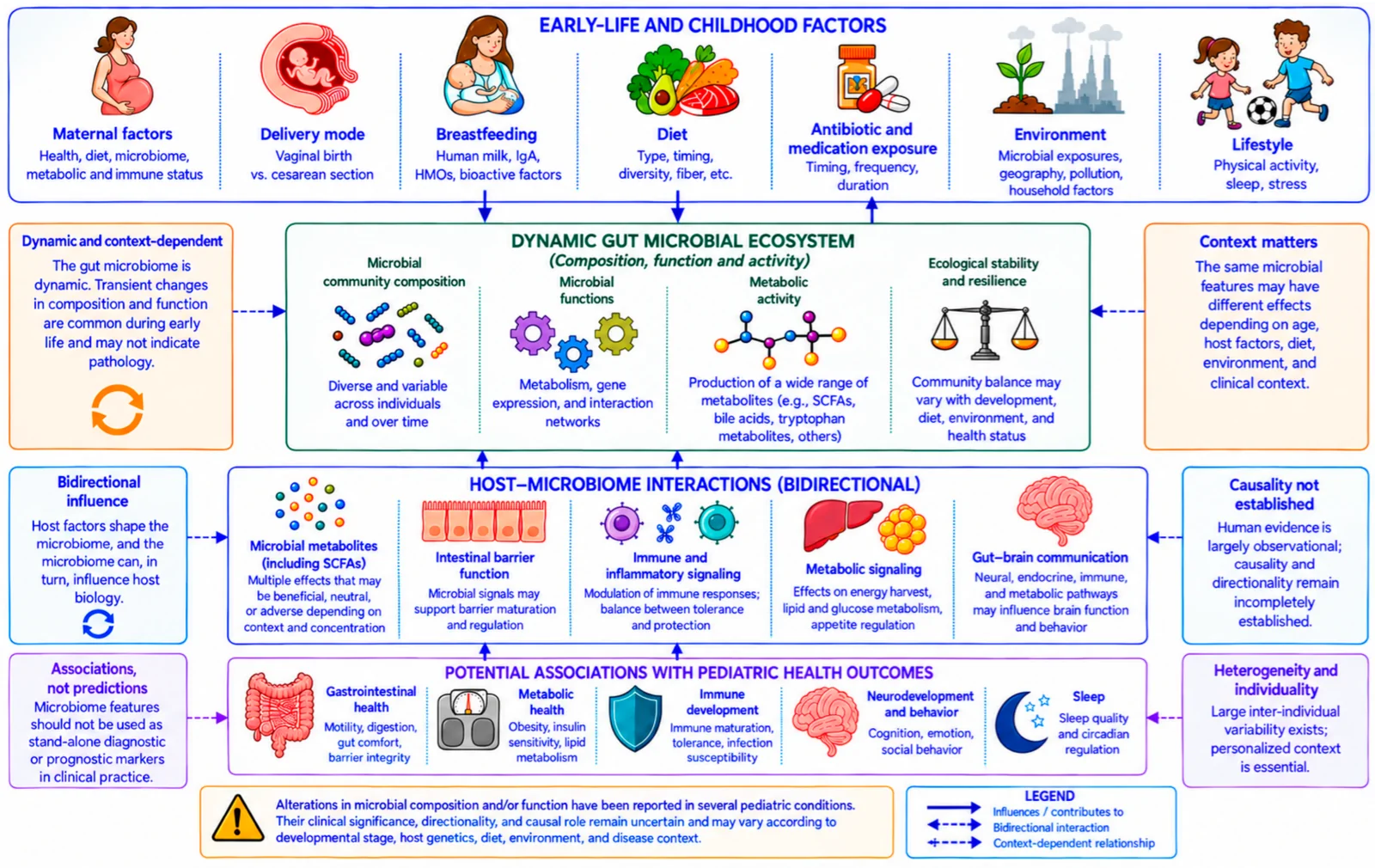
Summary infographic of the major themes, concepts, and relationships discussed during the symposium. Early-life and childhood factors, including maternal influences, delivery mode, breastfeeding, diet, antibiotic exposure, environmental exposures, and lifestyle, contribute to the development and function of the gut microbial ecosystem. Microbial composition and function may interact bidirectionally with host processes through microbial metabolites, intestinal barrier function, immune and metabolic signaling, and gut–brain communication. These interactions have been associated with gastrointestinal, metabolic, immunological, and neurodevelopmental outcomes, including sleep-related phenotypes. The figure represents a conceptual synthesis developed by the authors based on the topics and scientific concepts discussed during the symposium and is not reproduced from any individual presentation or external source. Generative AI was used as a visual design aid in the preparation of this figure. All scientific concepts, figure organization, content selection, interpretation, and final design were conceived, specified, reviewed, and approved by the authors, who take full responsibility for the accuracy and content of the figure.

**Figure 2:**
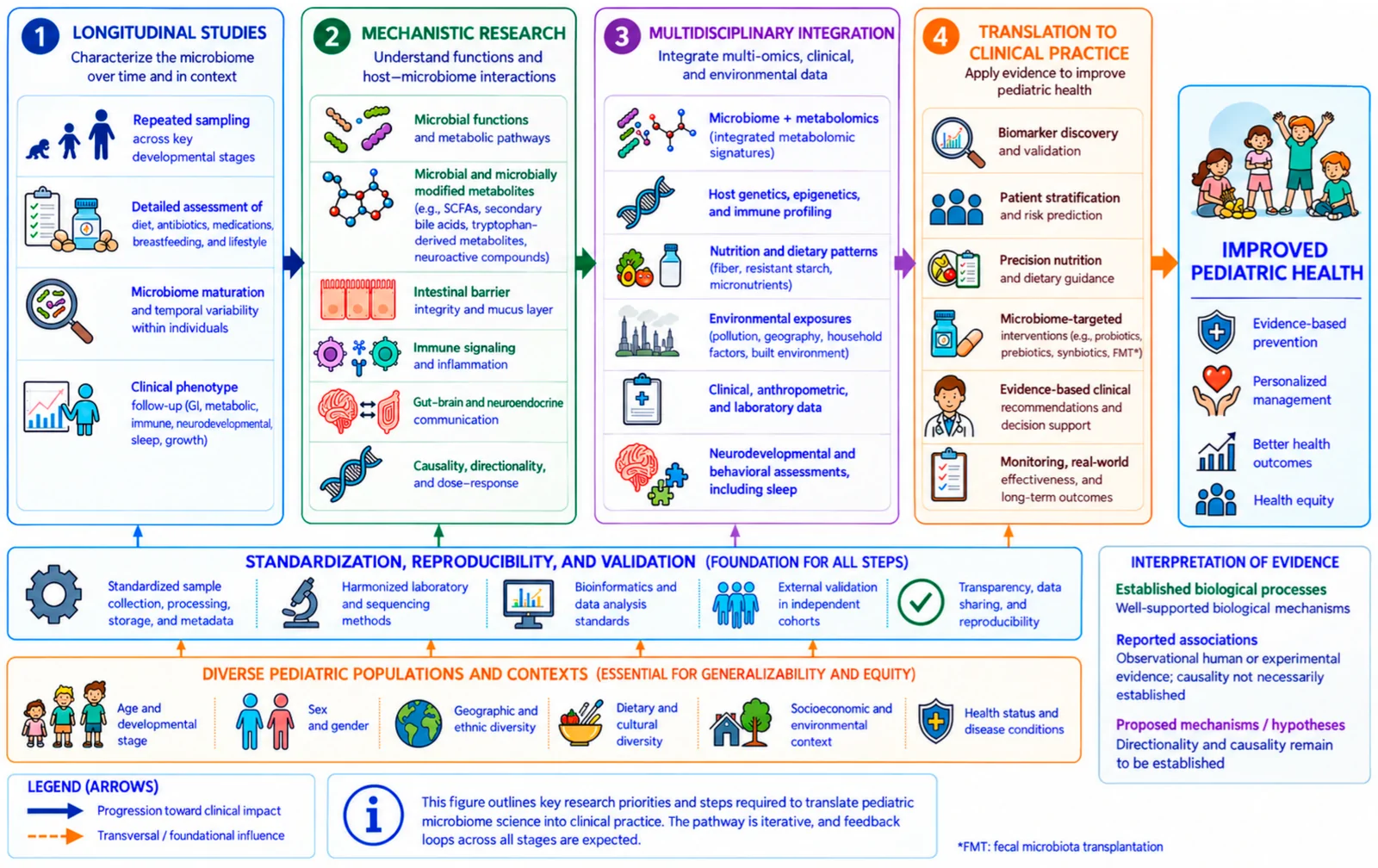
Advancing pediatric microbiome research through longitudinal studies, mechanistic research, multidisciplinary integration, and clinical practice. Standardization, reproducibility, external validation, and the inclusion of diverse pediatric populations are important considerations throughout this process. The framework differentiates findings supported by stronger biological evidence from reported associations and proposed mechanisms, emphasizing the need for further validation before microbiome-derived biomarkers or interventions can be incorporated into routine clinical practice. The figure was developed by the authors to summarize the main research priorities and future directions discussed during the symposium and is not reproduced from any individual presentation or external source. Generative AI was used as a visual design aid in the preparation of this figure. All scientific concepts, figure organization, content selection, interpretation, and final design were conceived, specified, reviewed, and approved by the authors, who take full responsibility for the accuracy and content of the figure.

**Table 1:** Overview of the scientific program represented in this Conference Report.

Session Theme	Lecture Title	Speaker	Affiliation
Microbiome During Lactation, Clinical Pediatrics and Immune Regulation	Probiotics in Pediatrics: From Evidence to Clinical Practice	José Francisco Cadena-León	National Pediatric Institute
	The Specialized Role of Maternal IgA Subclasses in the Configuration of the Microbiota and Neonatal Immunity	Leopoldo Santos-Argumedo	Center for Research and Advanced Studies
	Vertical Transmission of Microbiota and Metabolites During Lactation	Jaime García-Mena	Center for Research and Advanced Studies
Diet–Microbiome Interactions and Metabolic Health	The Microbiota: The Invisible Link Between Diet and Metabolic Diseases	Ana Isabel Burguete-García	National Institute of Public Health
	Starch Digestion and Gut Microbiota in Mexican Children: Metabolic Implications for the Risk of Insulin Resistance	Miguel Vázquez-Moreno	Mexican Institute of Social Security
The Microbiota–Gut–Brain Axis in Pediatric Health	The Microbiome–Gut–Mucosal–Immune System–Brain Axis and Autism Spectrum Disorder	Amapola De Sales-Millán	Autonomous Metropolitan University-Lerma
	Microbiota and Sleep Disorders in Pediatric Populations	Juan Carlos Ayala-García	National Institute of Public Health

**Table 2:** Strain-specific probiotic recommendations discussed in the ESPGHAN position paper.

Condition	Specific Strain/ Preparation	Population	Dose and Duration	ESPGHAN Recommendation/ Evidence
Acute gastroenteritis	*L. rhamnosus* GG	Children	≥10^10^ CFU/day; 5–7 days	May recommend; low certainty; weak recommendation. Reduced diarrhea duration, hospitalization, and stool output.
Acute gastroenteritis	*S. boulardii*	Children	250–750 mg/day; 5–7 days	May recommend; low certainty; weak recommendation. Reduced diarrhea duration.
Acute gastroenteritis	*L. reuteri* DSM 17938	Children	1 × 10^8^–4 × 10^8^ CFU/day; 5 days	May recommend; very-low certainty; weak recommendation. Reduced diarrhea duration.
Acute gastroenteritis	*L. rhamnosus* 19070-2 + *L. reuteri* DSM 12246	Children	2 × 10^10^ CFU of each strain/day; 5 days	May recommend; very-low certainty; weak recommendation. Reduced diarrhea duration.
Antibiotic-associated diarrhea prevention	*S. boulardii* or *L. rhamnosus* GG	Children with risk factors	≥5 × 10^9^ CFU/day; started with antibiotics	May recommend when risk factors are present; moderate certainty; strong recommendation.
Nosocomial diarrhea prevention	*L. rhamnosus* GG	Hospitalized children	≥10^9^ CFU/day; during hospitalization	May recommend; moderate certainty; weak recommendation.
Necrotizing enterocolitis prevention	*L. rhamnosus* GG; or *B. infantis* BB-02 + *B. lactis* BB-12 + *S. thermophilus* TH-4	Preterm infants	LGG: 1–6 × 10^9^ CFU/day; combination: 3–3.5 × 10^8^ CFU of each strain	May recommend, provided safety issues are met; low certainty; weak recommendation.
*H. pylori* infection	*S. boulardii*	Children receiving *H. pylori* therapy	Adjunctive use with eradication therapy	May recommend; very-low certainty; weak recommendation. May increase eradication rates and reduce gastrointestinal adverse effects.
Infantile colic	*L. reuteri* DSM 17938; *B. lactis* BB-12	Breastfed infants	10^8^ CFU/day; ≥21 days for *L. reuteri*; 21–28 days for BB-12	May recommend for breastfed infants; moderate certainty; weak recommendation. No recommendation for formula-fed infants due to insufficient evidence.
Functional abdominal pain disorders	*L. reuteri* DSM 17938; *L. rhamnosus* GG	Children	*L. reuteri*: 10^8^–2 × 10^8^ CFU/day; LGG: 10^9^–3 × 10^9^ CFU twice daily	May recommend; moderate certainty; weak recommendation. Reduction in pain intensity/frequency.
Functional constipation	—	Children	—	May not recommend probiotics as single or adjunctive therapy; moderate certainty; weak recommendation, due to lack of efficacy.
Inflammatory bowel disease	—	Children with Crohn disease or ulcerative colitis	—	No recommendation for or against probiotics studied so far because of insufficient evidence.

Note: ESPGHAN recommendations are strain- and indication-specific and should not be generalized to other probiotic strains, formulations, doses, or clinical settings. Evidence, certainty and recommendation strength vary by indication and strain [14].

## Data Availability

No new data were created or analyzed in this study.

## Supplementary Materials

The following supporting information can be downloaded at: https://www.mdpi.com/article/10.3390/microorganisms14102130/s1, Figure S1: Symposium banner; Table S1: Summary of selected key studies discussed during the symposium, including study design, methodology, key findings and main limitations.

## Abbreviations

The following abbreviations are used in this manuscript:

ASD	Autism Spectrum Disorder
IgA	Immunoglobulin A
sIgA	Secretory immunoglobulin A
IgA1	Subclass immunoglobulin A type 1
IgA2	Subclass immunoglobulin A type 2
IgG	Immunoglobulin G
IgM	Immunoglobulin M
SCFAs	Short-chain fatty acids
GABA	Gamma-aminobutyric acid
INP	Instituto Nacional de Pediatría
CINVESTAV	Centro de Investigación y de Estudios Avanzados
