# Supplementary material for: Pediatric Microbiota: Evidence and Future Perspectives—A Conference Report
Source: Microorganisms. 2026 Sep 22;14(10):2130. doi: 10.3390/microorganisms14102130 (PMC13707600; doi:10.3390/microorganisms14102130)
Supplement: Supplementary file 1 [file microorganisms-14-02130-s001.zip › microorganisms-4529820-supplementary.pdf]

**Supplementary Table S1. Summary of selected key studies discussed during the symposium, including study design, methodology, key findings, and main limitations.**

| Reference | Topic | Study design | Study methodology | Key findings | Main limitations |
|---|---|---|---|---|---|
| Stewart et al., 2018 [5] | Early-life microbiome development | Prospective longitudinal cohort study | Serial stool sampling in the TEDDY cohort; 16S rRNA sequencing and shotgun metagenomics were used to characterize microbiome maturation and assess maternal, environmental, dietary, and host covariates. | Identified distinct phases of gut microbiome maturation during early childhood and showed that breastfeeding, geography, delivery mode, antibiotic exposure, and other early-life factors influence microbial development. | Observational associations do not establish causality; the TEDDY cohort was enriched for children at genetic risk for type 1 diabetes, which should be considered when generalizing findings. |
| Szajewska et al., 2023 [14] | Probiotics in pediatric gastrointestinal disorders | ESPGHAN position paper / evidence-based review | Systematic reviews/meta-analyses and subsequently published RCTs were evaluated. Recommendations were formulated for well-defined probiotic strains and agreed through a consensus process. | Supports selected probiotic strains or combinations for specific pediatric gastrointestinal indications; recommendations are strain-, dose-, population-, and indication-specific. | Certainty of evidence varies across strains and indications; findings cannot be generalized to probiotics as a class, and additional studies are needed for several indications. |
| Baltierra-Uribe et al., 2021 [18] | Maternal IgA and Helicobacter pylori | Experimental immunological study | Colostrum IgA1 reactivity against *H. pylori* antigens was evaluated together with the ability of these antibodies to interfere with pathogen-induced changes in human epithelial cells. | Colostrum IgA1 recognized H. pylori antigens and prevented pathogen-associated cytoskeletal alterations in epithelial cells, supporting a potential protective role of maternal antibodies. | In vitro findings cannot establish the magnitude, persistence, or clinical effectiveness of protection against *H. pylori* infection in breastfed infants. |
| Corona-Cervantes et al., 2025 [19] | Maternal immunoglobulins and neonatal microbiota | Proof-of-concept molecular study | Flow cytometry coupled with 16S rRNA gene sequencing characterized IgA1-, IgA2-, IgM-, and IgG-bound bacterial communities in human colostrum and stool from breastfed neonates. | Maternal immunoglobulin classes displayed differential bacterial-binding patterns, and immunoglobulin-bound bacteria were detected in neonatal stool. | Proof-of-concept design limits generalization and does not establish whether immunoglobulin binding determines long-term microbiome composition, immune outcomes, or clinical effects. |
| Sánchez-Salguero et al., 2021 [20] | IgA2 and maternal-neonatal microbial interactions | Analytical, observational, prospective longitudinal study | Flow cytometry combined with IgA-Seq/16S rRNA sequencing was applied to maternal colostrum and neonatal fecal samples; diversity, composition, predicted functions, and source-tracking were assessed. | IgA2 preferentially targeted commensal bacterial genera shared between colostrum and neonatal feces, supporting a potential role in early microbial establishment. | Findings concern the first days of life; long-term persistence and clinical consequences were not established, and functional pathways were inferred rather than directly measured. |
| Díaz-Benítez et al., 2026 [27] | Bifidobacteria, B-vitamin intake, and metabolic health | Cross-sectional study | Anthropometric, biochemical, dietary, and microbiological data were evaluated in schoolchildren. Associations and interactions between intestinal bifidobacteria, dietary B vitamins, insulin-resistance markers, and serum lipids were assessed. | Combined exposure to intestinal bifidobacteria and B-vitamin intake was associated with indicators of insulin resistance and serum lipid levels, supporting diet-microbiota interactions in pediatric metabolic health. | Cross-sectional design precludes causal inference; species-level analyses involved a smaller subsample, and residual dietary and metabolic confounding cannot be excluded. |
| Ayala-García et al., 2024 [28] | Gut microbiota, central adiposity, and inflammation | Cross-sectional study | Five bacterial species, anthropometric variables, and serum cytokines were measured in 331 children. Regression, path analysis, and structural equation | *L. paracasei* was directly associated with lower IL-10; *S. aureus* and *A. muciniphila* showed indirect associations with adiponectin | Cross-sectional data limit temporal and causal inference; selected bacterial species rather than the complete microbial community were |

**Supplementary Table S1. Summary of selected key studies discussed during the symposium, including study design, methodology, key findings, and main limitations.**

| Reference | Topic | Study design | Study methodology | Key findings | Main limitations |
|---|---|---|---|---|---|
| | | | modeling assessed direct and waist-circumference-mediated associations. | through waist circumference, supporting interactions among microbiota, adiposity, and inflammation. | evaluated, and mediation models do not establish biological causality. |
| Orbe-Orihuela et al., 2022 [29] | Diet-dependent childhood obesity and gut microbiota | Cross-sectional study | Whole-metagenome shotgun sequencing was performed in 46 children. Dietary patterns were derived by principal component analysis, and regression models evaluated diet-microbiota interactions with BMI Z-score and adiposity measures. | Associations between microbial taxa and adiposity differed according to dietary pattern, supporting a diet-dependent relationship between gut microbiota and childhood obesity. | Small cross-sectional sample limits causal inference and generalizability; findings require replication in larger longitudinal pediatric cohorts. |
| Patloka et al., 2024 [24] | Diet, gut microbiome, and obesity | Narrative review | Synthesized evidence relating dietary patterns and nutrients, gut microbial composition, microbial metabolites, intestinal barrier function, inflammation, and obesity. | Highlights reciprocal relationships among diet, microbiome composition/function, microbial metabolites, intestinal barrier integrity, immune regulation, and obesity. | Narrative rather than systematic review; conclusions draw on heterogeneous human and experimental evidence, limiting causal interpretation and quantitative comparison. |
| Vázquez-Moreno et al., 2020 [30] | Starch digestion and childhood obesity | Case-control genetic/ metabolic association study with individual-participant meta-analysis | AMY1A/AMY2A copy numbers, serum amylase activity, dietary starch intake, and anthropometry were evaluated in Mexican children; AMY1A associations were additionally examined by meta-analysis. | Serum amylase activity was inversely associated with childhood obesity, particularly with medium/high starch intake; AMY1A copy number was associated with obesity in the larger meta-analysis. | Association-based design does not establish causality; diet may modify genotype/enzyme associations, and generalizability may depend on population genetic and dietary characteristics. |
| De Sales-Millán et al., 2024 [34] | Gut microbiota and autism spectrum disorder | Prospective comparative clinical study | Stool microbiota from children with ASD and neurotypical children was analyzed by V3-V4 16S rRNA sequencing, with taxonomic, sex-stratified, and predicted metabolic pathway analyses. | Differences were identified in selected bacterial genera and predicted metabolic pathways, including sex-associated patterns. | Relatively small sample; functional pathways were predicted from 16S data rather than directly measured by shotgun metagenomics or metabolomics. |
| Liu et al., 2019 [35] | Gut microbiota alterations in ASD | Systematic review | Multiple databases were searched according to PRISMA; culture-independent microbiome studies comparing ASD and controls were synthesized, with quality assessment and secondary functional analysis where data were available. | Reported recurring differences in microbial community structure and selected taxa between ASD and controls, together with predicted functional alterations. | Marked heterogeneity in diet, controls, GI symptoms, sampling, and microbiome methods produced inconsistent taxonomic findings and limited quantitative synthesis. |
| Leader et al., 2022 [36] | Gastrointestinal symptoms in ASD | Systematic review | Empirical studies assessing gastrointestinal symptoms in | GI symptoms, including constipation, diarrhea, and abdominal pain, were frequently | Considerable heterogeneity in study design and GI symptom measurement; standardized objective |

**Supplementary Table S1. Summary of selected key studies discussed during the symposium, including study design, methodology, key findings, and main limitations.**

| Reference | Topic | Study design | Study methodology | Key findings | Main limitations |
|---|---|---|---|---|---|
| | | | individuals with ASD younger than 18 years were systematically reviewed. | reported in children and adolescents with ASD. | assessments and larger prospective studies are needed. |
| Azari et al., 2024 [37] | Probiotics, prebiotics, and synbiotics in ASD | Systematic review and meta-analysis | Studies evaluating microbiome-targeted nutritional interventions in ASD were systematically identified and synthesized. | Microbiome-targeted interventions showed potential benefits for selected gastrointestinal and behavioral outcomes, but effects varied among studies. | Limited number and size of trials and substantial heterogeneity in formulations, doses, intervention duration, populations, and outcome measures. |
| de Groot et al., 2024 [38] | Sleep and early brain development | Narrative review | Integrated evidence on prenatal and postnatal sleep architecture, active/REM sleep, neural plasticity, functional connectivity, and methods for sleep assessment. | Highlights sleep as an active developmental process associated with synaptic remodeling, neural connectivity, and brain maturation. | Does not establish microbiome-mediated mechanisms or clinical interventions; further longitudinal and mechanistic studies are required. |
| Cryan et al., 2019 [39] | Microbiota-gut-brain axis | Comprehensive narrative review | Integrated preclinical and clinical evidence concerning neural, endocrine, immune, and metabolic pathways connecting the gut microbiota and central nervous system. | Established a broad framework for bidirectional microbiota-gut-brain communication, including microbial metabolites and neuroactive signaling pathways potentially relevant to sleep. | Much mechanistic evidence derives from animal and experimental models; translation to specific human pediatric outcomes remains limited. |
| Chen et al., 2022 [40] | Microbial metabolites, neurotransmitters, and gut-brain signaling | Narrative review | Reviewed microbiota-gut-brain interactions with emphasis on neurotransmitters and microbial modulation of GABAergic, serotonergic, tryptophan-related, and other neuroactive pathways. | Provides mechanistic plausibility for pathways through which intestinal microorganisms and their metabolites may influence neural function and sleep-related physiology. | Primarily mechanistic and disease-contextual evidence; direct causal evidence for microbiota-mediated sleep regulation in pediatric populations remains limited. |
| Park et al., 2026 [41] | Gut microbiome and insomnia | Cross-cohort meta-analysis | Five publicly available human fecal 16S rRNA case-control datasets (468 participants) were reprocessed using a unified pipeline; complementary statistical approaches and PICRUSt2 functional inference were applied. | Identified conserved gut microbial signatures associated with insomnia across independent cohorts, supporting reproducible microbiome-sleep associations. | Observational data do not establish causality; functional profiles were inferred rather than directly measured, and residual demographic, dietary, clinical, and medication-related confounding remains possible. |

**Abbreviations:** ASD, autism spectrum disorder; BMI, body mass index; ESPGHAN, European Society for Paediatric Gastroenterology, Hepatology and Nutrition; GABA, gamma-aminobutyric acid; GI, gastrointestinal; IgA, immunoglobulin A; IL-10, interleukin-10; PRISMA, Preferred Reporting Items for Systematic Reviews and Meta-Analyses; RCT, randomized controlled trial; REM, rapid eye movement; TEDDY, The Environmental Determinants of Diabetes in the Young.
